# Attitude of Medical and Dental Undergraduate Students Towards Learning of Communication Skills at a Private Medical Institute: A Cross-Sectional Study.

**DOI:** 10.31729/jnma.8671

**Published:** 2024-07-31

**Authors:** Pratisha Pradhan, Alok Kumar, Kabita Hada Batajoo, Pramita Shrestha, Trishna Shrestha, Sneha Pradhananga

**Affiliations:** 1 Department of General Practice and Emergency Medicine, KIST Medical College and Teaching Hospital, Imadole, Lalitpur, Nepal

**Keywords:** *attitude*, *communication skills*, *communication skill attitude scale*, *undergraduates*

## Abstract

**Introduction:**

Proper physician-patient communication has shown to impact patients' satisfaction, and better health outcomes. On the contrary, negative impacts of poor communication have been attributed as one of the causes of increasing workplace violence. It is imperative to identify the attitude of the students towards communication skill learning. The aim of the study was to find out the attitude of undergraduate students towards communication skill learning using the communication skill attitude scale.

**Methods:**

This cross-sectional study was carried out from October 2022 to July 2023 among undergraduate medical and dental students. Data was collected after obtaining the ethical approval from Institutional Review Committee (IRC number 2079/80/82) using a preformed proforma and the previously validated communication skills attitude scale questionnaire. Convenience sampling was done. The demographic details, educational characteristics, opinions regarding communication learning and median of positive and negative attitude scale scores were calculated using Statistical Package for the Social Sciences (SPSS).

**Results:**

The median (interquartile range) positive attitudes scale (PAS) and negative attitudes scale (NAS) scores were 52.00 (48.00-87.00) and 31.00 (28.00-34.00) respectively. The first year of undergraduates had higher PAS scores 56.00 (50.00-60.00) than final year 48.50 (44.25-55.00). The students who were in favour of ommunication skill learning during the clinical years of training had a higher PAS median score, 54.00 (49.00-58.00).

**Conclusions:**

The undergraduates had overall positive attitude towards communication skill learning but negative attitudes were also noted, with deterioration in the scores from first to final year of undergraduation, reiterating the importance of strengthening communication skill learning in the curriculum early-on in the study period.

## INTRODUCTION

Effective physician-patient communication is an essential skill of competency and has shown to significantly impact patients' satisfaction, care and better health outcomes. On the contrary, the negative impacts of poor communication are well documented and have been attributed as one of the core causes of increasing workplace violence.^1-3^ Promoting proper communication is one of the ways to lower negative impacts of poor physician-patient communication.^4^

Since 2008, communication skills (CS) has been embraced in the syllabus of Tribhuvan University (TU) in the undergraduate curriculum.^5^ Communication skills can be learned and taught, but negative attitudes of the students may hinder the success of these teaching programs.^5^

Despite inclusion in the curriculum, the attitude of medical students towards CS learning was not tested in our setting. Therefore, this study seeks to find the attitude of undergraduate students towards CS learning using the communication skill attitude scale (CSAS).^6^

## METHODS

This was a descriptive cross-sectional study conducted at KIST Medical College and Teaching Hospital (KIST MCTH), Imadol, Lalitpur, Nepal. It is a private medical institute, affiliated to Tribhuvan University (TU) offering a five-and-a-half-year undergraduation program in Bachelor of Medicine & Bachelor of Surgery (MBBS) and Bachelors in Dental Surgery (BDS) program. All the undergraduate medical and dental students of different phases, 769 students in total: Phase I: Year 1 and 2, phase II: Year 3, phase III: Year 4 and Year 5, and internship, present on the day of data collection and volunteering to participate were included in the study. Incomplete responses or non-participation were disregarded and did not bear any academic consequences.

The study was conducted from October, 2022 to July, 2023. Data was collected after ethical approval by the Institutional Review Committee (IRC number 2079/80/82) on January 5, 2023,. The printed proforma were handed over to two interns of emergency department (ED), who initiated the distribution of proforma among the undergraduate students in their respective lecture halls.

Convenience sampling was done. Written informed consent was obtained from each student The proforma consisted of a consent form (both in English and Nepali) and a printed questionnaire which contained four sections: title of the study, personal details, opinion regarding CS learning and the CSAS (in English). The 26-item-CSAS is a freely available, self-report questionnaire which has been validated widely.^7^ The scale possesses two subscales with 13 items on each subscale. Subscale I [4, 5, 7, 9, 10, 12, 14, 16, 18, 21, 22 (reversed score), 23 and 25] represents positive attitudes towards CS (PAS). Subscale II [1 (reversed score), 2, 3, 6, 8, 11, 13, 15, 17, 19, 20, 24 and 26] represents negative attitudes towards learning CS (NAS). Each statement is accompanied by a five-point Likert scale ranging from 1 (strongly disagree) to 5 (strongly agree). The score for each subscale may range from 13 to 65 where higher scores indicate stronger positive attitudes or stronger negative attitude towards CS learning.^6^

The collected information was entered in Microsoft excel and data analysis was done using Statistical Package for the Social Sciences (SPSS). Descriptive statistics were used to identify the personal characteristics of the participants in terms of number (n) and percentage (%). The mean with standard deviation (SD) of age and median with interquartile range (IQR) of PAS and NAS scores were tabulated.

## RESULTS

Out of 769 medical and dental undergraduates, 465 students duly filled out the questionnaire completely. Hence, the response rate was 60.46%. The reliability coefficient for each subscale of CSAS was calculated using Cronbach's alpha. Cronbach's alpha for PAS was found to be 0.74 which is indicative of acceptable internal consistency, and for NAS was found to be 0.50 showing poor internal consistency for these items. The mean age±SD was 22.22±1.92 years in medical undergraduates and 22.75±1.39 years in dental undergraduates (Table 1).

**Table 1:** Demographic and educational characteristics and median PAS and NAS scores of the participants (N=465)

Characteristics	Medical (n=381) n (%)	Dental (n=84) n (%)	PAS Median (IQR)^*^	NAS Median (IQR)^*^
Gender				
Male	206 (54.10)	9 (10.70)	52.00 (47.00-56.00)	32.00 (28.00-36.00)
Female	175 (45.90)	75 (89.30)	53.00 (48.00-58.00)	30.00 (27.00-33.00)
Address				
Koshi	29 (7.60)	7 (8.30)	54.00 (49.50-58.00)	29.00 (25.00-35.00)
Madhesh	39 (10.20)	5 (6.00)	54.00 (48.25-58.00)	32.50 (28.00-35.00)
Bagmati	241 (63.30)	59 (70.20)	52.00 (47.00-57.00)	30.00 (28.00-34.00)
Gandaki	18 (4.70)	3 (3.60)	50.00 (44.50-57.00)	29.00 (27.00-33.50)
Lumbini	30 (7.90)	8 (9.50)	52.50 (49.00-58.00)	33.00 (29.00-36.00)
Karnali	14 (3.70)	0 (0)	50.00 (47.25-56.00)	32.00 (30.00-33.75)
Sudurpaschim	10 (2.60)	2 (2.40)	54.50 (47.25-57.00)	30.00 (25.00-33.50)
Year of study				
First	77 (20.20)	0 (0)	56.00 (50.00-60.00)	32.00 (28.00-36.00)
Second	73 (19.20)	15 (17.90)	52.00 (47.00-58.00)	32.00 (29.00-36.00)
Third	80 (21.00)	15 (17.90)	54.00 (48.00-55.00)	32.00 (27.00-34.00)
Fourth	60 (15.70)	21 (25.00)	53.00 (48.00-59.00)	29.00 (27.00-32.50)
Final	32 (8.40)	8 (9.50)	48.50 (44.25-55.00)	31.50 (26.00-35.00)
Internship	59 (15.50)	25 (29.80)	50.00 (48.00-54.75)	30.00 (27.25-33.00)
Education (Schooling)				
Private	346 (90.80)	80 (95.20)	53.00 (48.00-57.00)	30.00 (27.00-34.00)
Government	35 (9.20)	4 (4.80)	50.00 (46.00-59.00)	33.00 (30.00-38.00)
Entry type				
Fresh batch	253 (66.40)	65 (77.40)	53.00 (48.00-57.00)	30.00 (28.00-34.00)
Old batch	128 (33.60)	19 (22.60)	52.00 (47.00-58.00)	31.00 (27.00-35.00)

* Interquartile range.

Outstanding CS was self-reported by 16 (4.20%) who also reported PAS median (IQR) of 56.50 (49.00-58.00). However, regarding written CS self-rating, 17 (4.46%) students who rated themselves as excellent had median (IQR) PAS of 49.00 (46.00-57.50), (Table 2).

**Table 2:** Self-rating of the participants (N=465)

Self-ratings	Medical (n=381) n (%)	Dental (n=84) n (%)	PAS Median (IQR)^*^	NAS Median (IQR)^*^
As a student				
Outstanding	16 (4.20)	0 (0)	56.50 (49.00-58.00)	31.00 (27.50-32.75)
Good	134 (35.20)	42 (50.00)	52.00 (47.00-56.00)	30.00 (27.00-34.00)
Average	179 (47.00)	35 (41.70)	54.00 (48.00-58.00)	31.00 (28.00-34.25)
Poor	52 (13.60)	7 (8.30)	51.00 (48.00-55.00)	32.00 (28.00-35.00)
Verbal communication skill				
Excellent	24 (6.30)	5 (6.00)	54.00 (46.00-56.50)	32.00 (26.00-34.00)
Good	153 (40.20)	32 (38.10)	52.00 (48.00-56.00)	31.00 (28.00-34.00)
Average	143 (37.50)	44 (52.40)	53.00 (47.00-58.00)	30.00 (27.00-34.00)
Poor	61 (16.00)	3 (3.50)	52.00 (49.00-56.00)	31.00 (27.25-35.00)
Written communication skill				
Excellent	17 (4.50)	0 (0.00)	49.00 (46.00-57.50)	32.00 (27.50-35.50)
Good	122 (32.00)	29 (34.50)	53.00 (47.00-56.00)	31.00 (28.00-34.00)
Average	188 (49.30)	49 (58.40)	52.00 (48.00-58.00)	30.00 (27.00-34.00)
Poor	54 (14.20)	6 (7.10)	53.00 (49.00-56.00)	32.00 (28.25-35.00)

* Interquartile range; PAS: Positive Attitudes Scale; NAS: Negative Attitudes Scale

There were 82 (21.52%) medical students and 1 (1.19%) dental students with prior exposure to CS lessons and showed median (IQR) PAS of 54.00 (48.00-59.00) and NAS of 30.00 (27.00-34.00), (Table 3). CS was thought to be a learnable course by 326 (85.56%) medical students and by 30 (35.71%) dental students and 302 (79.27%) medical students and 34 (40.48%) dental students thought it should be taught as part of their curriculum (Table 3). Among medical students 308 (80.84%) wished to attend such classes. Despite this, 299 (78.48%) of them had not attended any CS lessons during their medical study period.

**Table 3:** Opinions regarding CS learning of participants (N=465)

Opinion	Medical (n=381) n (%)	Dental (n=84) n (%)	PAS Median (IQR)^*^	NAS Median (IQR)^*^
Have you attended any communication skill lesson in medical study period?			
Yes	82 (21.50)	1 (1.20)	54.00 (48.00-59.00)	30.00 (27.00-34.00)
No	299 (78.50)	83 (98.80)	52.00 (48.00-56.25)	31.00 (28.00-34.00)
Do you think communication skill learning is important for a physician?			
Yes	364 (95.60)	33 (39.30)	53.00 (48.00-57.50)	31.00 (27.00-34.00)
No	4 (1.00)	0 (0)	50.00 (45.25-58.50)	32.00 (22.75-39.75)
May be	13 (3.40)	51 (60.70)	49.00 (46.00-52.75)	30.00 (29.00-33.75)
Do you think communication skill learning should be taught as part of curriculum?			
Yes	302 (79.30)	34 (40.40)	54.00 (49.00-58.00)	31.00 (27.00-34.00)
No	24 (6.30)	4 (4.80)	46.00 (41.25-50.50)	34.00 (28.00-37.00)
May be	55 (14.40)	46 (54.80)	49.00 (46.50-54.00)	30.00 (28.00-33.50)
Do you think you can actually learn communication skills?			
Yes	326 (85.60)	30 (35.70)	54.00 (48.00-58.00)	30.00 (27.00-34.00)
No	7 (1.80)	0 (0.0)	47.00 (44.00-51.00)	33.00 (31.00-39.00)
May be	48 (12.60)	54 (64.30)	49.00 (46.00-53.00)	31.00 (29.00-34.00)
Do you wish to attend a communication skill lesson?			
Yes	308 (80.80)	33 (39.20)	54.00 (48.50-58.00)	30.00 (27.00-33.00)
No	22 (5.80)	5 (6.00)	45.00 (39.00-51.00)	35.00 (29.00-39.00)
May be	51 (13.40)	46 (54.80)	49.00 (46.00-55.00)	31.00 (29.00-36.00)

* Interquartile range; PAS: Postitive Attitudes Scale; NAS: Negative Attitudes Scale

Highest PAS was seen in phase II medical students 80 (21.00%) and Phase I in dental students 15 (17.86%) with median score (IQR) of 54.00 (50.25-55.00) and 54-.00 (50.00-59.00) respectively (Table 4).

**Table 4:** Median PAS and NAS scores among medical and dental undergraduates according to phases (n=465)

Undergraduates	n (%)	PAS Median (IQR)^*^	NAS Median (IQR)^*^
**Medical (n=381)**			
Phase I	150 (39.37)	53.50 (48.00-59.00)	32.00 (28.00-36.00)
Phase II	80 (21.00)	54.00 (50.25-55.00)	32.00 (27.00-34.00)
Phase III	92 (24.15)	52.50 (47.00-58.75)	31.00 (27.00-34.00)
Internship	59 (15.49)	52.00 (48.00-57.00)	29.00 (25.00-33.00)
**Dental (n=84)**			
Phase I	15 (17.86)	54.00 (50.00-59.00)	30.00 (29.00-36.00)
Phase II	15 (17.86)	47.00 (42.00-50.00)	31.00 (29.00-34.00)
Phase III	29 (34.52)	52.00 (47.50-54.50)	29.00 (25.50-32.00)
Internship	25 (29.76)	49.00 (46.00-49.00)	30.00 (30.00-33.00)

* Interquartile range; PAS: Postitive Attitudes Scale; NAS: Negative Attitudes Scale

## DISCUSSION

The current study shows that there was not much variation in the PAS and NAS scores among the undergraduates in terms of demographic factors (age and provinces) and educational characteristics (schooling and entry type). It may be due to the increasing equality, development and globalization that the groups are becoming more homogeneous. However, a previous study at Universities of Nottingham and Leicester, and a review article based on 31 randomized studies, 38 open effect studies and 14 descriptive studies, showed that female students have higher PAS scores and lower NAS scores.^8,9^ Like our study results, Shankar et al. also had inconclusive results regarding gender and attitude towards CS learning.^10^

In this study, the total median (IQR) PAS score was 52.0 (48.00-57.00) and NAS score was 31.00 (28.00 34.0). The first-year undergraduates had higher PAS scores, 56.00 (50.00-60.00), which reiterates the fact that CS learning can be started from the initial years of undergraduate medical studies. This initiation could be pivotal in bringing out skillful and successful future physicians. Previous studies have definitely highlighted the importance of CS among pre-clinical and clinical undergraduates separately^4,11-13^, but this study gives a broader picture of when the possible best time of improvising the curriculum could be.

At a private medical institute, in Aruba, a similar study was done where there were no CS courses as part of the curriculum in the institute and due to the overall positive attitude towards CS the researcher and team planned to start CS learning in the institution right from the first semester, with opportunities for supervised practice during early clinical exposure and hospital observer-ship with standardized patients.^10^

A pre-post study was conducted by C. Rees and C. Sheard in 2002 which indicate that CS should be taught using experiential methods and within an appropriate clinical context.^14^ Despite the current emphasis on CS training, studies have also reported that the PAS scores do not increase significantly.^15,16^ In our study, most undergraduates had strong positive attitudes toward learning CS, however, curriculum planners should not lose sight of negative attitudes and appropriate measures need to be taken to minimize them because the negative attitudes may belittle the importance of the learning of CS.

The current study shows the attitude towards learning CS among different phases of undergraduation and how the deterioration in their positive attitudes have occurred from the first year to internship. The possible reason behind this could be because the TU curriculum does not bear any academic consequences for the lack of CS practices. Self-ratings as a student, regarding verbal and written CS have been mentioned in this study and most students believe they are average students, which is similar to study done in Pokhara.16studies in Nepal are lacking. Although a standard rating of their CS abilities was not done in this study, their self-rating gives us a clue that there is room for improvement. Studies suggest that students with poor CS value the opportunities offered by CS training courses more highly.^9^

Lack of proper communication has often been mentioned as one of the motives for increased violence among health care professionals and health facilities in Nepal.^17^ All around the world, medical students and institutes focus more on clinical skills over CS and the situation in Nepal is no different. In this study, 79.27% of medical undergraduates believe that CS should be taught as part of the curriculum while only 40.48% of dental undergraduates believe the same. The students who were in favour of CS learning during the clinical years of training had a higher PAS score of 54.00 (49.00-58.0). Following similar research, Soltani et al. also concluded that increased CS in physicians can lead to patients' satisfaction and better outcome and that it is necessary to add CS theories in the curriculum.^18^

This study has also included dental undergraduates as they are equally medical undergraduates as M.B.B.S students. 78.48% of medical undergraduates and 98.81% of dental undergraduates had not attended any CS lesson in their study periods. This gives a picture of their previous knowledge regarding CS. On the contrary, a study carried out among the third and fourth semester MBBS students at Manipal College of Medical Sciences (MCOMS), Pokhara showed 87.80% had undergone CS courses during their clinical years.^16^ Likewise, 88.00% third and fifth year MBBS students at Chitwan Medical College (CMC) also had CS course during their clinical years.^11^

This study shows 95.54% of medical and 39.29% of dental students thought good doctor-patient communication to be an important physician skill. The low percentage among dental students could be because medical students need to have more prolonged communication with the patients as compared to the dental students, yet the results clearly show their higher positive attitude towards learning CS among the first and second year (phase I) dental students, 54.0 (50.00-59.00). Hence the curriculum needs to be strengthened among dental undergraduates as well.

A similar comparison was made by Nourein et al.in Saudi Arabia with no differences in self-rating their attitudes towards.^19^ Studies regarding CS learning among dental studies have been done separately in Nepal, but results have not been compared to medical undergraduates as in our study.^20,21^

The current study was conducted at KIST MCTH, a private institute including a small sample size. Hence, the results of the study cannot be generalised to all undergraduate medical students throughout the country. Prior exposure to any formal training on CS is not a criterion for exclusion in this study. Thus, their attitude towards CS learning may be biased positively or negatively depending on their prior training experience. The study also reports the selfperception of the participants and does not include actual communicating abilities. This study only reports the attitude towards CS and does not however include any implementation of any training. There was student diversity with respect to demographic characteristics and this could be one of the reasons for the low alpha coefficient for the negative attitudes.

## CONCLUSIONS

The undergraduates have overall positive attitude towards CS learning but negative attitudes were also noted, with deterioration in the scores from first to final year of undergraduation, reiterating the importance of strengthening CS learning in the curriculum early-on in the study period. Undergraduates with exposure to CS training/lessons had more positive attitude towards learning CS in this study, thus a motivation to involve more of CS trainings in the undergraduate curriculum.
